# Mechanisms of Endothelial Dysfunction in Resistance Arteries from Patients with End-Stage Renal Disease

**DOI:** 10.1371/journal.pone.0036056

**Published:** 2012-04-26

**Authors:** Leanid Luksha, Peter Stenvinkel, Folke Hammarqvist, Juan Jesús Carrero, Sandra T. Davidge, Karolina Kublickiene

**Affiliations:** 1 Division of Obstetrics & Gynecology, Karolinska Institutet, Karolinska University Hospital, Department of Clinical Science, Intervention & Technology, Stockholm, Sweden; 2 Division of Renal Medicine, Karolinska Institutet, Karolinska University Hospital, Department of Clinical Science, Intervention & Technology, Stockholm, Sweden; 3 Division of Surgery, Karolinska Institutet, Karolinska University Hospital, Department of Clinical Science, Intervention & Technology, Stockholm, Sweden; 4 Department of Obstetrics and Gynecology, University of Alberta, Edmonton, Alberta, Canada; University of Illinois at Chicago, United States of America

## Abstract

The study focuses on the mechanisms of endothelial dysfunction in the uremic milieu. Subcutaneous resistance arteries from 35 end-stage renal disease (ESRD) patients and 28 matched controls were studied *ex-vivo*. Basal and receptor-dependent effects of endothelium-derived factors, expression of endothelial NO synthase (eNOS), prerequisites for myoendothelial gap junctions (MEGJ), and associations between endothelium-dependent responses and plasma levels of endothelial dysfunction markers were assessed. The contribution of endothelium-derived hyperpolarizing factor (EDHF) to endothelium-dependent relaxation was impaired in uremic arteries after stimulation with bradykinin, but not acetylcholine, reflecting the agonist-specific differences. Diminished vasodilator influences of the endothelium on basal tone and enhanced plasma levels of asymmetrical dimethyl L-arginine (ADMA) suggest impairment in NO-mediated regulation of uremic arteries. eNOS expression and contribution of MEGJs to EDHF type responses were unaltered. Plasma levels of ADMA were negatively associated with endothelium-dependent responses in uremic arteries. Preserved responses of smooth muscle to pinacidil and NO-donor indicate alterations within the endothelium and tolerance of vasodilator mechanisms to the uremic retention products at the level of smooth muscle. We conclude that both EDHF and NO pathways that control resistance artery tone are impaired in the uremic milieu. For the first time, we validate the alterations in EDHF type responses linked to kinin receptors in ESRD patients. The association between plasma ADMA concentrations and endothelial function in uremic resistance vasculature may have diagnostic and future therapeutic implications.

## Introduction

Adverse cardiovascular events are common complications of end-stage renal disease (ESRD) and these patients are more likely to die from cardiovascular disease (CVD) than from kidney failure [1]. Although the underlying mechanisms that predispose ESRD patients to higher risk of CVD are incompletely understood, morphological and functional abnormalities of the endothelium may play an important role [2]. Endothelial dysfunction is considered an early marker of CVD [3], which facilitates the progress of atherosclerosis [4] and contributes to the development of hypertension through the enhancement of vascular resistance [5]. Thus, studies aimed to at investigating the mechanisms of endothelial dysfunction in ESRD are of importance and may provide a means to ameliorate cardiovascular complications and introduce novel treatment strategies.

Our current knowledge of endothelial dysfunction in ESRD is mainly based on findings from animal models [6], circulating plasma markers [7] and *in-vivo* assessments in the human forearm [8]. Although few attempts have been made to estimate endothelial function in resistance arteries of ESRD patients [9]–[11], the mechanisms of endothelial dysfunction need further clarification. NO deficiency has been considered as a principal event leading to endothelial dysfunction in the uremic milieu [12]. However, the contribution of NO to endothelium-dependent control of vascular tone is inversely associated with caliber of arteries. Another vasodilator, known as endothelium-derived hyperpolarizing factor (EDHF), seems to act as a predominant mediator of endothelium-dependent dilatation in resistance-size arteries. EDHF type responses are characterized by endothelium-dependent hyperpolarization that occurs due to direct electrical coupling via myoendothelial gap junctions (MEGJs) and/or the release of different mediators [13]. The role of EDHF in endothelial maintenance has been introduced as a back-up mechanism during NO deficiency. However when deprivation of EDHF occurs, this may further aggravate endothelial dysfunction leading to enhanced blood pressure and impaired blood flow to target organs [13].

Studies concerning the detailed mechanisms of endothelial dysfunction in resistance arteries with a focus on the relative contribution of NO and EDHF in ESRD are scarce. Current data on the contribution of EDHF to endothelium-dependent relaxation of resistance arteries in kidney failure are mainly based on animal studies and characterized by explicit heterogeneity [6], [14]–[18]. To the best of our knowledge, the only study that has investigated the relative contribution of EDHF *vs.* NO to acetylcholine (ACh)-induced-relaxation in ESRD patients has reported an impairment in NO-mediated responses but an unchanged, or even increased, role of EDHF as assessed by forearm blood flow [19].

In this study, we hypothesized that endothelial dysfunction in resistance arteries of incident dialysis patients is not only restricted to impairment in production and/or bioavailability of NO, but EDHF type responses may also be affected by uremic milieu. To test this hypothesis we isolated arteries from subcutaneous fat to segregate pharmacologically the relative impairments in NO and EDHF type responses that confer endothelial dysfunction in ESRD. Targeted pathways of endothelial dysfunction were assessed using basal and receptor-dependent stimulation of endothelium-derived vasodilators, expression of endothelial NO synthase (eNOS), prerequisites for MEGJ, and associations between endothelium-dependent responses and plasma levels of endothelial dysfunction surrogate markers.

## Results

### Participants

Age, gender, and smoking status were similar between the groups. The body mass index was lower in ESRD patients *vs.* controls. Plasma levels of asymmetrical dimethyl L-arginine (ADMA), soluble vascular cell adhesion molecule-1 (sVCAM-1), interleukin-6, pentraxin-3, high sensitivity C-reactive protein (hsCRP) and lipoprotein(a) and triglycerides were elevated in ESRD. No differences in blood pressure or total cholesterol were observed between the groups (Table 1).

**Table 1:** Baseline characteristics of ESRD patients and controls.

Parameters	ESRD (n = 35)	Controls (n = 28)
Age (years)	57±13	54±14
Males n (%)	24 (69)	21 (75)
Body mass index (kg/m^2^)	24.1±3.3*	27.6±3.7
Systolic blood pressure (mmHg)	144±21	138±17
Diastolic blood pressure (mmHg)	86±11	84±11
Total cholesterol (mmol/L)	4.6±1.2*	5.1±1.0
Triglycerides (mmol/L)	1.6* (0.8–5.7)	1.3 (0.7–19)
Lipoprotein(a) (mg/L)	409 (50–2572)*	156 (50–802)
S-albumin (g/L)	34.9±3.4*	38.4±3.4
S-Creatinine (µmol/L)	620 (249–1069)*	78 (55–100)
Interleukin-6 (pg/ml)	5.0 (1.9–16.8)*	1.5 (0.4–17.1)
hsC-reactive protein (mg/L)	1.9 (0.2–24.9)*	1.4 (0.4–13.9)
Pentraxin-3 (ng/ml)	1.2 (0.5–8.3)*	0.6 (0.1–2.3)
Fibrinogen (g/L)	4.8±1.3*	3.2±1.3
Glomerular filtration rate (ml/min)	12±3*	89±3
Asymmetric Dimethylarginine (µmol/L)	0.6±0.1*	0.5±0.1
Soluble VCAM-1 (ng/ml)	1295 (637–1980)*	588 (368–830)
Soluble ICAM-1 (ng/ml)	223 (138–404)	231 (161–363)
Diabetes mellitus n, (%)	10 (29)	0
CVD, n, (%)	13 (37)	0
Antihypertensive treatment, n, (%)	33 (94)	0
Statin treatment, n, (%)	15 (43)	0

* , *P*<0.05.

### Vascular function

In total, 84 subcutaneous arteries with internal diameter of 209±6 µm were dissected from 35 ESRD patients and 71 arteries with internal diameter of 224±7 µm were dissected from 28 controls (*P*>0.05). There was no difference in the magnitude of pre-constriction 3 µmol/L norepinephrine between the groups (2.5±0.2 mN/mm^2^ ESRD *vs.* 2.7±0.2 mN/mm^2^ controls).

### Endothelium-dependent relaxation

**Figure 1:**
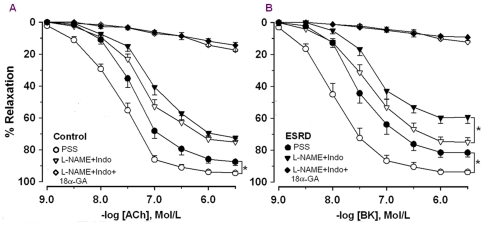
Concentration response curves to acetylcholine (ACh, A) and bradykinin (BK, B). Responses in physiological salt solution (PSS) and after incubation with *N* ^ω^-nitro-l-arginine methyl ester plus indomethacin alone (L-NAME+Indo) or together with 18α-glycyrrhetinic acid (L-NAME+Indo+18α-GA) in arteries from ESRD patients (n = 32 for ACh and n = 22 for BK) and controls (n = 23 for ACh and n = 17 for BK). * ESRD *vs.* controls, *P*<0.05.

In controls, ACh and bradykinin (BK) caused relaxation of arteries with similar magnitude (Figure 1). However, arteries were more sensitive to BK *vs.* ACh (pEC_50_, here and in the following text: BK 7.9±0.1 *vs.* ACh 7.7±0.1, *P* = 0.01). NOS/cyclooxygenase (COX) inhibition reduced endothelium-dependent relaxation (Figure 1) and the sensitivity to agonists became similar (ACh: 7.2±0.1 *vs.* BK: 7.3±0.1, *P* = 0.5).

Relaxation and sensitivity to both agonists was attenuated in ESRD *vs.* controls (Figure 1). In contrast to the controls, the sensitivities of ESRD arteries in PSS were similar between the agonists (ACh: 7.3±0.1 vs. BK: 7.4±0.1, *P* = 0.2).

**Figure 2:**
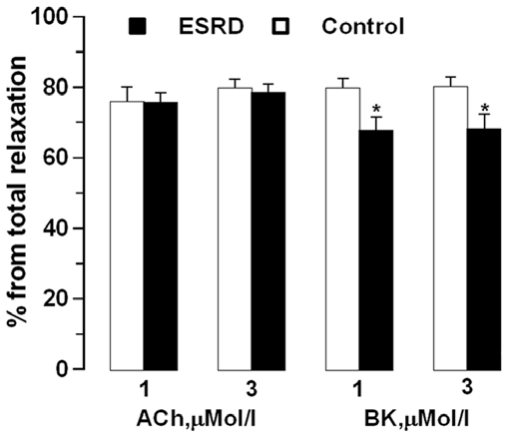
The relative contribution of endothelium-derived hyperpolarizing factor (EDHF). Contribution of EDHF in arteries from ESRD patients and controls in response to acetylcholine (ACh) and bradykinin (BK). * ESRD *vs.* controls, *P*<0.05.

After NOS/COX inhibition relaxation and sensitivity to ACh and BK were attenuated in ESRD (Figure 1). The concentration-response curves after NOS/COX inhibition were shifted to the right in ESRD *vs.* controls (Figure 1). However, the maximal EDHF type relaxation was reduced in ESRD *vs.* controls in response to BK but not to ACh (BK: *P* = 0.003; ACh: *P* = 0.09). Moreover, the relative contribution of EDHF was reduced in ESRD *vs.* controls in response to BK but not to ACh (Figure 2).

An inhibitor of gap junctions (18-α-glycyrrhetinic acid, 18-αGA) markedly reduced EDHF type relaxation in response to both agonists (Figure 1). There was no difference in residual relaxation after incubation with 18-αGA along with NOS inhibitor, *N* ^ω^-nitro-L-arginine-methyl ester (L-NAME) and COX inhibitor, indomethacin (Indo) between ESRD *vs.* controls (Figure 1). The relative contribution of MEGJs to EDHF type responses was similar between ESRD and controls independently of the agonist used (ACh, 1 µmol/l: 86±4 ESRD (n = 16) *vs.* 80±6 controls (n = 12), *P* = 0.7; BK, 1 µmol/l: 85±5 ESRD (n = 13) *vs.* 87±3 controls (n = 8), *P* = 0.9).

In order to eliminate the possible interference of co-morbidities, the responses to agonists before and after NOS/COX inhibition in ESRD patients without diabetes mellitus (DM) and CVD were compared with those of controls. In response to BK we observed similar results as above (Figure 1B). In contrast to the whole ESRD group, in arteries from ESRD without DM and CVD, ACh-induced relaxation was reduced in PSS as compared to controls but similar after NOS/COX inhibition (ACh after NOS/COX inhibition: 7±0.1 ESRD without DM and CVD (n = 18) *vs.* 7.2±0.1 controls (n = 23), *P* = 0.1).

### Transmission electron microscopy (TEM)

**Figure 3:**
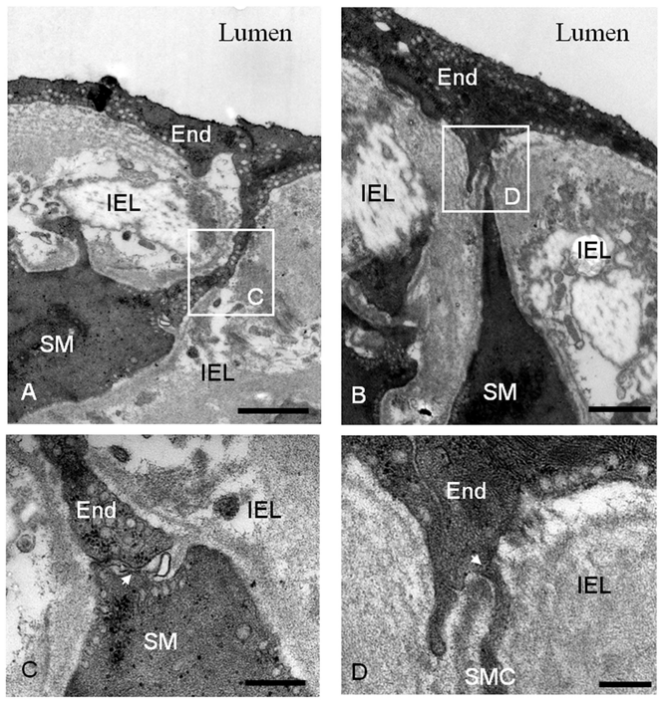
Transmission electron images of arteries from ESRD patients. The lower magnification pictures (A,B) show an overview of the vascular wall with endothelium (End) and smooth muscle (SM) being separated by the internal elastic lamina (IEL). The areas denoted by the boxes are magnified and show the sites of intercellular contacts that could be considered as prerequisites of myoendothelial gap junctions (C, D). The width of the gap is ∼20 nm (C, arrow), ∼11 nm (D, arrow). Bar: (A) 2 µm; (B) 3 µm; (C) 0.1 µm; (D) 0,2 µm.

Analysis of TEM images focused on morphological prerequisites for the gap junctions between EC and smooth muscle cells (SMC) in arteries from ESRD patients and controls (n = 3). The main criteria for identification of MEGJs was the presence of the characteristic pentalaminar membrane structure at points of cell to cell contact, where the central region had a higher electron opacity than the inner parts and distance between the EC and SMC plasma membranes was around 3.5 nm [20]. TEM images showed the presence of long protrusions (up to 4.5 µm) from both ECs (Figure 3A) and SMCs (Figure 3B) penetrating the internal elastic lamina and forming close contacts with each other (Figure 3C and 3D). Although the observed EC-SMC contacts did not fulfill all criteria for the characteristic pentalaminar structures, they could be considered as prerequisites for MEGJs.

### Endothelium-independent relaxation

**Figure 4:**
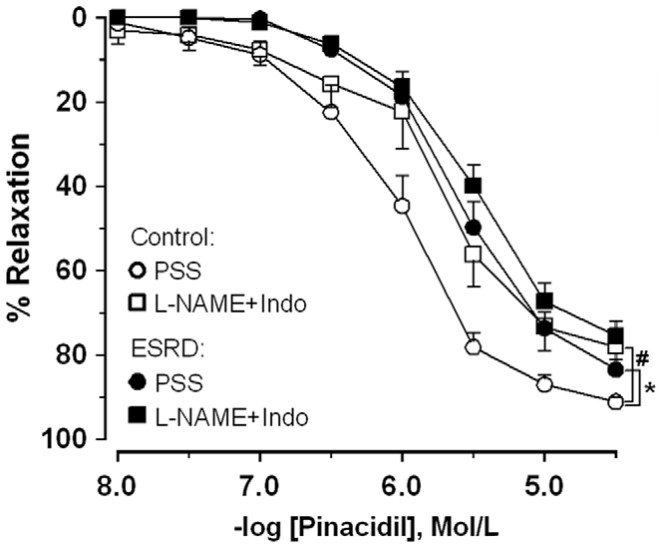
Concentration response curves to pinacidil. Responses in arteries from ESRD patients (n = 10) and controls (n = 7) in physiological salt solution (PSS) and after incubation with *N* ^ω^-nitro-l-arginine methyl ester plus indomethacin (L-NAME+Indo). * ESRD *vs.* controls, *P*<0.05; ^#^ before *vs.* after incubation with L-NAME+Indo, *P*<0.05.

Endothelium-independent relaxation to NO donor sodium nitroprusside (SNP) was similar between the groups (pEC_50_: 6.0±0.1 ESRD (n = 17) *vs.* 6.2±0.2 controls (n = 13), *P* = 0.6). In ESRD patients pinacidil-induced responses were blunted as compared to controls (6.1±0.1 ESRD *vs* 5.7±0.1 controls, *P* = 0.01, Figure 4). NOS/COX inhibition attenuated the response in controls but not in ESRD (6.1±0.1 PSS *vs* 5.8±0.1 L-Name+Indo, *P* = 0.03 controls; 5.7±0.1 *vs* 5.6±0.1, *P* = 0.3 ESRD, respectively, Figure 4). There was no difference between ESRD and controls in their responses to pinacidil after NOS/COX inhibition (Figure 4).

### Influence of the endothelium-derived factors on basal tone and expression of eNOS

**Figure 5:**
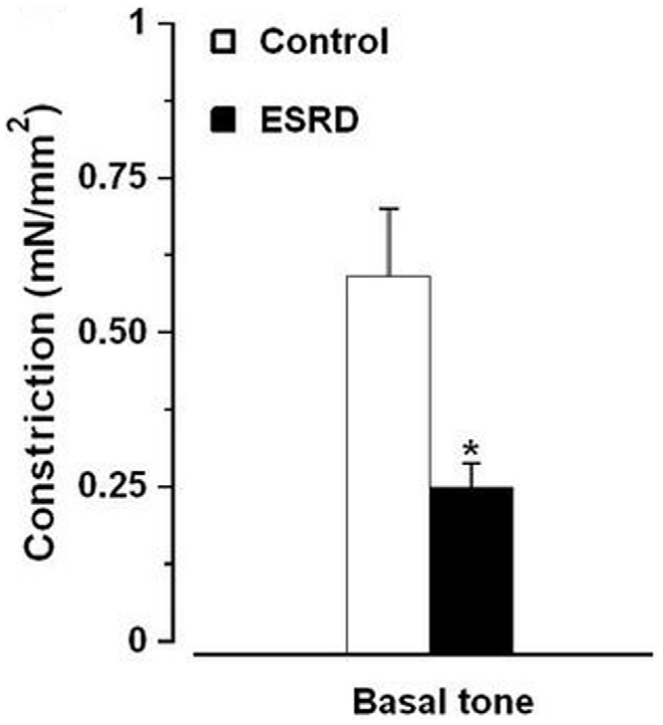
Contractile response to NOS/COX inhibitors of arteries from controls (n = 26) *vs.* ESRD patients with (n = 32). *, *P*<0.05 ESRD *vs.* controls.

**Figure 6:**
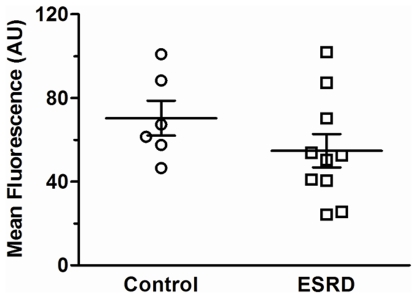
Endothelial nitric oxide synthase (eNOS) expression in arteries from controls (n = 6) and ESRD patients (n = 10).

NOS/COX inhibitors induced constriction of arteries from both ESRD and control groups. This constriction was reduced in ESRD *vs.* controls (Figure 5). Exclusion of the patients with DM and CVD from ESRD group did not change the outcome (Figure 5; 0.28±0.1 mN/mm^2^ ESRD without DM and CVD (n = 17) *vs.* 0.59±0.1 mN/mm^2^ controls (n = 26), *P* = 0.02). There was no difference in eNOS expression in ESRD *vs.* controls (Figure 6).

### Associations between endothelium-dependent responses and plasma markers of endothelial dysfunction

**Figure 7:**
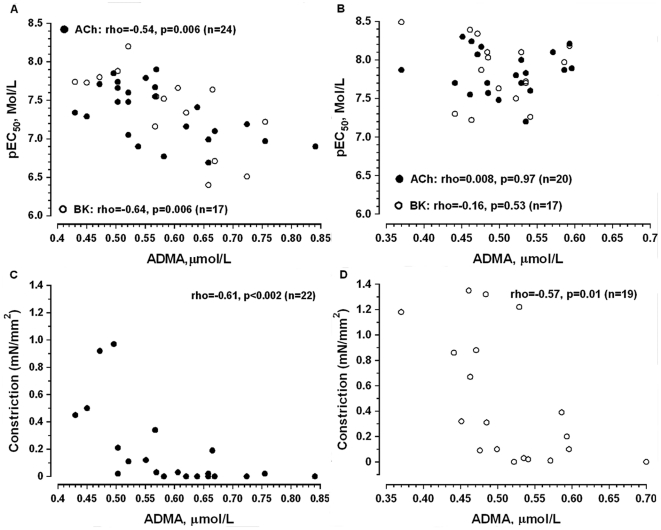
Spearman rank correlation between plasma levels of asymmetrical dimethyl L-arginine (ADMA, µmol/L) and artery sensitivity to endothelium-dependent vasodilators (pEC_50_, A, B) or vasoconstriction in response to NOS/COX inhibition (L-NAME+Indo, C, D) in ESRD patients (A, C) and controls (B, D).

The sensitivity of arteries to ACh and BK was negatively associated with plasma levels of ADMA in ESRD (Figure 7A) but not in controls (Figure 7B). There was no association between ADMA and vascular sensitivity to the endothelium-dependent agonists after NOS/COX inhibition in both groups. Similarly, sensitivity to SNP was not associated with ADMA levels (data not shown).

In contrast to the agonists-induced relaxation, constriction in response to NOS/COX inhibition was negatively associated with plasma levels of ADMA in both ESRD and controls (Figure 7C and 7D).

No relation was found between resistance artery function and other surrogate markers of endothelial dysfunction (soluble intercellular adhesion molecule-1 (sICAM-1) and sVCAM-1) in ESRD and controls (data not shown).

## Discussion

In this *ex-vivo* study of human uremic resistance arteries we describe, for the first time, the relative role of endothelium-derived factors, agonist-specific differences and associations between endothelial function and surrogate plasma markers of endothelial dysfunction. We show that reduced EDHF type responses contribute markedly to endothelial dysfunction in ESRD. Impaired EDHF type responses in ESRD were detected with endothelium-dependent agonist BK but not ACh. Thus, we suggest that changes in signal transduction from endothelial receptors towards generation and/or transformation of hyperpolarization to the smooth muscle are differently affected by uremic toxins with a predominant impact on those mediated by kinin receptors. Diminished vasodilator influence of the endothelium on basal tone of SMCs along with enhanced plasma levels of ADMA indicates an impairment in NO-mediated control of arterial tone in ESRD. While the eNOS expression and the contribution of MEGJs to EDHF type responses appeared to be unaltered in uremic arteries, the upstream machinery of both endothelial pathways (i.e. NO and EDHF) were impaired. Since relaxation in response to NO donor or hyperpolarizing agent pinacidil (after NOS/COX inhibition) were similar between the groups, we confirm that the endothelium is the main target of uremic environment, whereas functional capacity of the vascular smooth muscle appeared to be rather tolerant. In accordance with our previous study [11] we corroborate a central role of the uremic milieu in the genesis of endothelial dysfunction. The present study also shows that among measured plasma markers of endothelial dysfunction only ADMA was strongly associated with the magnitude of endothelial dysfunction in uremic resistance arteries. Thus, our findings provide novel insights into the mechanisms of endothelial dysfunction in resistance circulation of ESRD patients.

The pattern of impairment of EDHF type responses after BK but not ACh stimulation in uremic arteries emphasizes the agonist-specific mechanisms of endothelial dysfunction in this toxic milieu. We speculate that conventional and/or disease-specific risk factors may differently affect kinin and muscarinic receptors and/or their regulatory pathways. For example, endothelial dysfunction in atherosclerosis appears to be receptor-specific, involving the muscarinic receptors with relative sparing of the kinin receptor pathways. Abnormal reactivity of epicardial coronary arteries during physiologic stress is better represented by BK and not by ACh responses [21]. Moreover, differences exist between BK- and ACh- induced relaxation of the mesenteric arteries from spontaneously hypertensive rats at different ages, suggesting a more detrimental effect of increased blood pressure on BK-induced vasorelaxation [22], while selective impairement of endothelium-dependent relaxation to ACh but not BK is observed in isolated small omental arteries from women with preeclampsia [23]. Thus, prior conclusions based only on the vascular effects of one agonist (i.e. ACh) should be considered with caution. In contrast to previous studies, in which only ACh was tested [9]–[10], our more comprehensive analysis of pathways involved in endothelial dysfunction of resistance arteries in ESRD patients revealed an impairment of EDHF contribution coupled with stimulation of kinin receptors.

In contrast to our findings on heterogeneity of mechanisms of EDHF type responses in preeclampsia [24]–[25], the present data revealed MEGJs as a common pathway of EDHF in both groups. Hence, the compensatory response for preservation of endothelial function via the flexibility of mechanisms behind EDHF type responses seems to be lacking in ESRD. Since the contribution of EDHF and MEGJ to ACh-induced relaxation was similar between the two experimental groups, it is unlikely that impairment at the level of MEGJs could be linked with kinin receptors. Therefore, it can be speculated that alterations in BK-induced EDHF type responses in uremic resistance arteries may reside in the signaling sequence extending from B_2_-kinin receptors to the activation of Ca^2+^-dependent K^+^-channels (K_ca_-channels) generating hyperpolarization of the endothelium with following transformation to the smooth muscle via MEGJ. Further studies are warranted to clarify the involvement of particular endothelial K_ca_-channels in alterations of BK-induced EDHF type responses in this patient group.

Despite the fact that EDHF normally does not act through the K_ATP_-channels [13], pinacidil-induced responses allowed us to assess the general mechanism of relaxation induced by hyperpolarization due to outward K^+^ currents at the level of the smooth muscle [26]. Moreover, an animal study of renal failure suggested that alterations in smooth muscle K^+^-channels could be involved in reduced endothelium-dependent hyperpolarization [16]. In our study, relaxation to pinacidil was reduced in ESRD *vs.* controls but this difference disappeared after NOS/COX inhibition, which opposes the findings in the animal study [16]. Most likely basal NO had a potentiating effect on pinacidil-induced relaxation in controls but not in ESRD patients. While in general relaxation induced by K_ATP_-channels openers has yet been considered endothelium-independent [27], the potentiating effects of endothelium-derived factors has been reported before [25], [28].

Impaired endothelial influence on pinacidil-induced responses may further support our data about reduced basal release of endothelium-derived factors in ESRD. Indeed, NOS/COX inhibitors induced smaller constriction in uremic *vs.* control arteries. As basal vascular tone is to a large extend NO-dependent [29], our data implies a reduction in basal production of NO in ESRD. In contrast, a previous study reported increased basal NO production in the forearm of hemodialysis patients [30]. The inconsistent results may be caused by different methodology, and selection of patients. Recently, we demonstrated the lack of NO contribution to shear stress responses in subcutaneous uremic arteries [11]. In the current study, differences in sensitivity between BK and ACh, depending from NOS/COX inhibition in controls but not in ESRD, indicated on distinct NO contribution to agonist-induced relaxation between the two groups. Moreover, the negative correlation between serum ADMA levels and relaxation to ACh and BK in ESRD but not in controls further supports the impaired contribution of NO to agonists-induced responses in uremia.

Multiple mechanisms may lead to NO deficiency in renal failure [12], [31]. A decreased bioavailability of NO due to increased pro-oxidative environment has been suggested [11]. Reduced expression of eNOS has been linked to a decreased NO production in an animal model of kidney failure [32]. Studies on EC cultures have shown that erythrocytes [33] or sera fractions enriched with advanced glycation end products [34] from uremic patients may directly affect expression and activity of eNOS. However, we failed to find any difference in eNOS protein expression between uremic and control arteries. Moreover, an elevated vascular expression but unchanged activity of eNOS was demonstrated in radial arteries of ESRD patients [35]. Nevertheless, such observations might indicate that unchanged or even increased expression of eNOS in the vascular wall could not guarantee a sufficient NO bioavailability in ESRD patients. We therefore speculate that endothelial dysfunction along with unchanged expression of eNOS, may represent a potential outcome of a reduced ability of the enzyme to generate NO via eNOS uncoupling with following decrease in NO bioavailability in uremic resistance arteries.

ADMA may serve as a feasible candidate to link the uremic environment with endothelial dysfunction in the resistance vasculature. Indeed, ADMA, as an endogenous inhibitor of eNOS that accumulates when renal function declines [36], it has been shown to induce eNOS uncoupling either via substrate reduction or via direct effect on eNOS catalysis and, as a consequence, eNOS will generate superoxide radicals instead of NO [37]. On the other hand, we cannot exclude the possibility that the effect of ADMA on endothelial superoxide generation may be due to the activation of other enzymatic sources of superoxide radicals such as NADPH oxidases. ADMA may also stimulate renin angiotensin system, and ADMA induced impairement of NO-mediated function due to increased superoxide production has been shown to occur via activation of of Ang II-NADPH oxidase pathway in isolated small vessels from rats [38]. In animal models of experimental diabetic nephropathy, AngII induced activation of NADPH oxidase and eNOS uncoupling serves as the major source of superoxide, and the blockade of AngII signaling ameliorates eNOS uncoupling by increased tetrahydrobiopterin levels with following restoration of NO bioavailability and improved glomerular hemodynamics [39]–[40].

Although, an association between elevated ADMA levels and endothelium-dependent dilatation in the forearm was previously reported [41], we are the first to show an association between ADMA and endothelium-dependent relaxation in uremic resistance vasculature. The inverse correlation between ADMA and changes in basal tone after NOS/COX inhibition in both experimental groups support *in-vivo* results demonstrating that ADMA increases vascular resistance in ESRD patients [42] and in healthy humans [43]. Taken together our data endorse the proposal that elevated ADMA may act as a potential mechanism behind the impaired NO-dependent control of uremic resistance artery tone.

On the other hand, adhesion molecules sICAM-1 and sVCAM-1, two purported biomarkers of endothelial dysfunction, did not correlate with changes in basal tone nor with responses to endothelium-dependent agonists. As ADMA is a potentially modifiable risk factor, future interventional studies primarily focusing on acute and long term L-arginine supplementation [44] or regulation of dimethylarginine dimethylaminohydrolase activity that confers the intracellular ADMA concentrations [45] are of interest.

In summary, by studying the mechanisms of endothelial dysfunction in uremic resistance arteries we were able to dissect the impairment in basal and agonist-specific effects of two endothelium-derived vasodilators - EDHF and NO. For the first time, we provided evidence of impaired EDHF type responses particularly linked to kinin receptors in ESRD. The current results support our previous findings that NO plays a critical role in uremic endothelial dysfunction in resistance circulation. The observation that preserved responses of smooth muscle to pinacidil and NO-donor indicated a decisive role of malfunctions within the endothelium and tolerance of vasodilator mechanisms to the uremic retention products at the level of smooth muscle. As this study showed an association between circulating plasma ADMA concentrations and endothelial dysfunction in uremic resistance vasculature, our findings may have diagnostic and future therapeutic implications.

## Materials and Methods

### Participants

The study was approved by the Ethical Committee at Karolinska University Hospital and conducted according to the principles expressed in the Declaration of Helsinki. All participants involved in the research gave written informed consent prior to enrollment.

Subcutaneous fat biopsies were obtained from 35 ESRD patients at the time of peritoneal dialysis catheter insertion. Only patients starting dialysis treatment were included. Exclusion criteria were acute infection, vasculitis or liver disease at the time of evaluation. Control tissue was obtained from 28 age-matched volunteers without renal, mental or diabetic disease who underwent hernia repair (n = 17) or laparoscopic cholecystectomy (n = 11).

### Baseline Laboratory and Clinical Assessments

Clinical history of CVD or DM was obtained from medical records. CVD was defined as the presence of ischemic cardiac disease, peripheral vascular disease and/or cerebrovascular disease. Ongoing medication was collected from medical charts. Glomerular filtration rate was estimated by the mean of creatinine- and urea clearances in ESRD patients, whereas cystatin-C estimated glomerular filtration rate in the controls. Fasting venous blood samples were taken. Plasma and serum were stored at −70°C pending further analyses. Serum interleukin-6 was measured on an Immulite® analyzer (Siemens Medical Solution Diagnostic, Los Angeles, CA, USA). Serum concentrations of albumin, creatinine, lipids and hsCRP were measured routinely. ADMA was assessed in serum by ELISA assays (DLD Diagnostika GMBH, Germany). Concentrations of pentraxin-3, sICAM-1 and sVCAM-1 were measured in serum (ELISA assays from R&D systems, USA).

### Vascular function

Arteries were isolated and mounted on two stainless steel wires (25 µm in diameter) in the organ baths of a four-channel wire myograph (model 610, Danish Myo Technology; Aarhus, Denmark) as described previously [25]. Arteries collected from patients with ESRD we refer as “uremic arteries".

Once a sustained, steady contraction to norepinephrine (3 µmol/L) was attained, the concentration-response curves to the endothelium-dependent vasodilators ACh and BK (1 nmol/L to 3 µmol/L) or SNP (10 nmol/L to 100 µmol/L), NO-donor and an opener of ATP-sensitive K^+^-channels (K_ATP_-channels), pinacidil (10 nmol/L to 100 µmol/L), were obtained. Arteries were then incubated for 20 min with NOS inhibitor (300 µmol/L) and COX inhibitor, Indo (10 µmol/L). Subsequently, arteries were pre-constricted again and second concentration-response curve for endothelium-dependent agonists was obtained. The term “EDHF" used in this study refers to the L-NAME+Indo-insensitive component of endothelium-dependent vasodilatation. The level of increased resting tone of the arteries after incubation with L-NAME+Indo was considered as an index of vasoactive properties of the endothelium, reflecting a basal release of endothelium-derived vasoactive factors. To evaluate the contribution of gap junctions in EDHF type responses, the concentration-response curves to ACh and BK were constructed after 15 min co-incubation with 18-αGA (100 µmol/L) in the presence of L-NAME+Indo.

### Fluorescence immunohistochemistry

Freshly isolated arteries where cryopreserved in optimal cutting temperature compound on dry ice. Transverse 8 µm cryosections were prepared and mounted onto slides, air-dried, and stored at −80°C. For immunostaining, cryosections were incubated for 1.5 hr at room temperature with the mouse polyclonal anti-eNOS antibody (1∶250, BD Biosciences 610296). Incubation with the secondary goat anti-mouse antibody (Invitrogen, Alexa fluor 488 A11001) was done for 1 hr in the dark. Glass coverslips were mounted with Vectashield H-1200 Mounting Kit (Vector Laboratories). Stained sections were examined immediately under fluorescence microscope. All images presented are in (×100) magnification.

### Transmission electron microscopy

Artery segments were fixed as described previously [24]–[25]. Serial transverse, ultra-thin sections (approximately 50–80 nm) were cut. The series consisted of 3–5 sections. For each artery such series were repeated three times after an interval of 10 µm. Sections were examined in a Tecnai 10 transmission electron microscope at 80 kV and digital images were captured.

### Chemicals

The composition of PSS was (in mmol/L): NaCl 119, KCl 4.7, CaCl_2_ 2.5, MgSO_4_ 1.17, NaHCO_3_ 25, KH_2_PO_4_ 1.18, EDTA 0.026, and glucose 5.5. The chemicals were obtained from Sigma, St. Louis. To prepare stock solution, the substances were dissolved in distilled water. Indo and pinacidil were dissolved in ethanol and 18α-GA was dissolved in DMSO. Pilot studies showed that the solvents used had no effect upon vascular responses at their final concentrations.

### Data analysis

In figures, results are expressed as mean ± SEM. In tables, normally distributed variables are expressed as mean ± SD, and non-normally distributed variables as medians and interquartile ranges. Baseline characteristics of the patients and arteries used and staining were analysed by conventional parametric and non-parametric methods. The isometric force developed by artery segment during application of vasoactive compounds was calculated using Myodata (Danish Myo Technology, Denmark) and expressed as mN/mm^2^. Relaxation was expressed as a percentage of the pre-constriction. In order to visualize the relative contribution of EDHF or MEGJs, a percentage of the relaxation was calculated after pre-incubation with L-NAME+Indo or L-NAME+Indo+18α-GA and related to the full response in PSS or after pre-incubation with L-NAME+Indo. Negative log concentration (in mol/l) required to achieve 50% of the maximum response (pEC_50_) was calculated by nonlinear regression analysis (BioDataFit 1.02). ANOVA was used to compare concentration-response curves before and after incubation with different inhibitors. Spearman's rank correlation was used to determine the associations between artery sensitivity (pEC_50_) to endothelium-dependent vasodilators and plasma markers of endothelial dysfunction. Significance was taken at the 5% level for all comparisons. All statistical analyses were performed with STATISTICA (v.10.0, StatSoft, Uppsala, Sweden).
